# Association between life’s essential 8 and testosterone deficiency in men: NHANES 2011–2016

**DOI:** 10.3389/fendo.2024.1394383

**Published:** 2024-06-03

**Authors:** Min Cai, Jinzao Chen

**Affiliations:** Department of Cardiology, The First Hospital of Putian City, Putian, Fujian, China

**Keywords:** life’s essential 8, testosterone deficiency, NHANES, cardiovascular disease, restricted cubic spline

## Abstract

**Background:**

Serum testosterone is intrinsically associated to cardiovascular disease. Our aim is to explore the relationship between the recently updated cardiovascular health measurement, known as Life’s Essential 8 (LE8), and the prevalence of testosterone deficiency (TD) in adult males in the United States.

**Methods:**

Study data was obtained from the National Health and Nutrition Examination Survey (NHANES) from 2011 to 2016. A weighted multivariate logistic regression model was applied to evaluate the correlation between LE8 and testosterone deficiency. Restricted Cubic Spline (RCS) was employed to explore its non-linear relationship. In addition, a stratified analysis was conducted.

**Results:**

The final analysis included 2332 participants from NHANES from 2011 to 2016. After adjusting for confounding factors, the odds ratios (ORs) and 95% confidence intervals (CIs) for testosterone deficiency in participants with moderate and higher LE8 scores compared to the lowest LE8 scores were 0.59 (0.38–0.92) and 0.38 (0.19–0.76), respectively. The results of subgroup analysis showed that LE8 score was significantly associated with TD among young and middle-aged participants.

**Conclusion:**

A lower LE8 score is related to a higher incidence of testosterone deficiency, especially in young and middle-aged men. Further research is necessary to explore the potential mechanisms between them.

## Introduction

Testosterone, a pivotal hormone that primarily defines male physiology, is synthesized in the labyrinthine interstices of the Leydig cells. This hormone, quintessential in its roles, orchestrates a symphony of biological processes essential to men’s health, including sexual function, heart vigor, energy flux, mind clarity, and skeletal strength (1–3). As such, a decrease or deficiency in male serum testosterone levels can result in multiple organ dysfunction. Moreover, beyond decline and erectile dysfunction, low testosterone levels are also linked to and may exacerbate related metabolic diseases such as depression and osteoporosis, collectively referred to as testosterone deficiency syndrome (4–6). Given its prevalence, testosterone deficiency is a widespread condition, with the incidence rate notably increasing with age. Projections indicate that this situation will worsen with the increase of average lifespan in the coming decades (7). Consequently, the global alarm over dwindling testosterone levels is intensifying.

In 2010, the American Heart Association (AHA) unveiled Life’s Simple 7 (LS7), a guideline designed to elevate cardiovascular health (CVH) across the populace. This directive advocates for a holistic approach: it emphasizes a nutritious diet, abstaining from tobacco, maintaining a healthy body mass index (BMI), engaging in sufficient physical activity, and ensuring blood pressure, fasting blood sugar, and cholesterol levels remain within desirable ranges (8). To further improve the evaluation of CVH, the AHA recently introduced Life’s Essential 8 (LE8), which advances the original LS7 by incorporating sleep quality indicators and refining the scoring algorithm (9, 10).

Studies indicate that serum testosterone levels are intricately linked with cardiovascular health, suggesting that bolstering CVH could be a potent strategy to mitigate the impact of serum testosterone deficiency (11, 12). While prior research has predominantly employed LE8 as a metric for assessing cardiovascular health, it has revealed a significant association between the two. However, to date, no one has previously utilized LE8 to evaluate serum testosterone levels. Therefore, our study sought to delve into the nexus between LE8 and serum testosterone deficiency, aiming to bridge the existing knowledge gap.

## Materials and methods

### Data and sample sources

NHANES is a systematic cross-sectional health survey project that utilizes a stratified, multi-stage, and probabilistic clustering design. This cross-sectional analysis employed data from the 2011–2015 NHANES cycle, with exclusion criteria as follows: (1) individuals who were under 20 years old and Female (n=51,278), (2) those with missing LE8 and serum testosterone data (n=2293), (3) those with fasting weight (wtsaf2yr) =0 (n=3154) and (4) those with covariate-related missing values (n=308). In the end, our investigation encompassed a cohort of 2332 participants (**Supplementary Figure 1**).

### Measurement of LE8

The LE8 scoring system integrates four lifestyle behaviors—diet, physical activity, nicotine exposure, and sleep duration—with four health indicators: body mass index (BMI), non-high-density lipoprotein cholesterol, blood sugar, and blood pressure. The detailed methodology for computing the LE8 score, utilizing NHANES data, has been thoroughly outlined in prior publications and can be found in **Supplementary Table 1** (9). Each of the eight CVH indicators receives a rating on a scale of 0 to 100. The total LE8 score is determined by the unweighted average of these eight indicators, with participants achieving high CVH classified as having an LE8 score of 80–100; moderate CVH is defined as ranging from 50 to 79; and low CVH is considered as 0–49.

### Assessment of serum testosterone level

According to the guidelines of the American Association of Urologists (AUA), symptoms of TD are identified by serum testosterone levels below 300ng/dL (13, 14). To minimize biological variability, blood samples were drawn between 8:30 am and 11:30 am after a night of fasting. Subsequently, specimens were frozen on dry ice for immediate use or long-term storage at -70°C. To measure the total serum testosterone levels, a competitive electrochemiluminescence immunoassay was utilized on the Elecsys automated analyzer to determine the serum testosterone levels of these samples, with a minimum linear limit of 0.75 ng/dL. For more detailed experimental information, please refer to the following link: https://wwwn.cdc.gov/Nchs/Nhanes/2013–2014/TST_H.html.

### Covariates

In our study, the covariates include several factors previously demonstrated or presumed to affect the relationship between Life’s Essential 8 and serum testosterone, including age, gender, race, education, marital status, poverty income ratio (PIR), alcohol consumption, smoking history, hypertension, and diabetes (DM). The detailed classification of these covariates is presented in **Table 1**.

**Table 1:** Baseline characteristics of participants from the NHANES, 2011–2015.

Characteristics	Total (N=2332)	Non-testosterone deficiency (n=1837)	Testosterone deficiency (n=495)	*P* value
Age, years, mean (SD)	47.91±0.45	46.94±0.56	51.83±0.77	<0.0001
Race, n (%)				0.57
Non-Hispanic White	1054 (70.82)	807 (70.18)	247 (73.39)	
Non-Hispanic Black	427 (9.30)	343 (9.59)	84 (8.09)	
Mexican American	298 (7.77)	240 (7.95)	58 (7.04)	
Other Hispanic	225 (5.44)	180 (5.60)	45 (4.80)	
Other race	328 (6.67)	267 (6.67)	61 (6.68)	
Education, n (%)				0.58
High school grad or equivalent	1210 (56.04)	956 (56.77)	243 (53.05)	
Less than high school	477 (14.77)	365 (14.45)	112 (16.07)	
Some college or above	645 (29.19)	505 (28.78)	140 (30.87)	
PIR, n (%)				0.24
<1.3	680 (19.51)	532 (19.68)	148 (18.81)	
1.3-3.5	307 (10.84)	243 (10.66)	64 (11.57)	
>3.5	1345 (69.65)	1062 (69.66)	283 (69.61)	
BMI, n (%)				<0.0001
<25	631 (24.85)	573 (28.52)	58 (10.00)	
25-29.99	884 (38.12)	738 (40.90)	146 (26.89)	
≥ 30	817 (37.02)	526 (30.58)	291 (63.10)	
Moderate recreational activities, n (%)				0.18
No	1364 (55.46)	1053 (54.56)	311 (59.13)	
Yes	968 (44.54)	784 (45.44)	184 (40.87)	
Smoking status, n (%)				<0.0001
Never	1087 (48.18)	869 (49.48)	218 (42.91)	
Former	738 (31.42)	538 (28.67)	200 (42.54)	
Current	507 (20.40)	430 (21.85)	77 (14.55)	
Alcohol drinking, n (%)				0.002
Never	175 (7.02)	139 (7.01)	36 (7.10)	
Former	406 (14.20)	287 (12.28)	119 (21.97)	
Mild	966 (42.35)	761 (41.79)	205 (44.59)	
Moderate	272 (13.19)	221 (14.15)	51 (9.29)	
Heavy	513 (23.24)	429 (24.76)	84 (17.05)	
Hypertension, n (%)				<0.0001
No	1300 (59.55)	1087 (62.29)	213 (48.43)	
Yes	1032 (40.45)	750 (37.71)	282 (51.57)	
Diabetes, n (%)				<0.0001
No	1808 (83.10)	1503 (86.80)	305 (68.12)	
Yes	524 (16.90)	334 (13.20)	190 (31.88)	

BMI, body mass index; RIP, ratio of family income to poverty.

### Statistical analyses

Acknowledging the intricate sampling framework of NHANES, our analysis meticulously incorporated suitable sample weights throughout the study. The chi-square test and Kruskal-Wallis test were utilized to assess if significant differences existed between the different LE8 groups (divided into three categories) and TD. A multivariate logistic regression model was applied to investigate the independent association between LE8 and TD. Within this framework, Model I did not include adjustments for covariates; Model II was specifically adjusted for age, race, education and family annual income; Model III, expanding on Model II, further included adjustments for BMI, moderate recreational activity, smoking, alcohol drinking, hypertension and diabetes. Restricted cubic spline regression was used to explore potential non-linear relationships between the LE8 score, its subscale scores, and TD. To examine subgroups that might be affected by demographic differences, stratified analysis was conducted based on different age, age group, race/ethnicity, poverty rate, and education level. A *p*-value of less than 0.05 was considered to indicate statistical significance.

## Results

### Baseline characteristics

A total of 2332 eligible participants participated in the NHANES 2011–2016 cycle. LE8 is divided into three groups based on scores: low (LE8<50), moderate (50 ≤ LE8<80), and high (LE8 ≥ 80). The overall prevalence of testosterone deficiency is 21.2%, and participants with testosterone deficiency are typically observed to tend to be older, exhibit a higher BMI, and are more likely to smoke and drink. Additionally, they are more likely to have diabetes and hypertension (**Table 1**).

Association between LE8 scores and testosterone deficiency.

**Figure 1:**
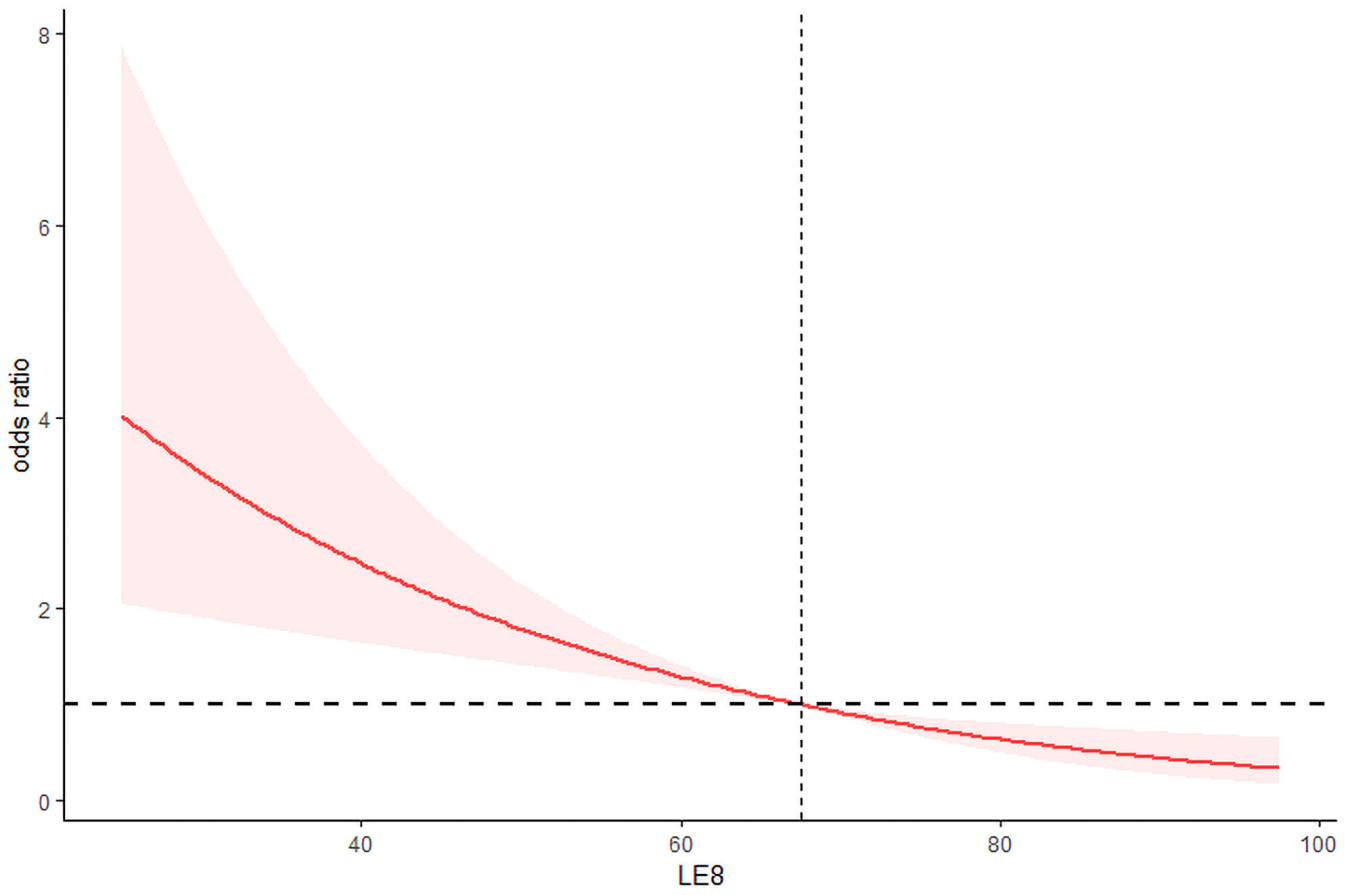
Restricted cubic spline for relationship of LE8 with risk of life’s essential 8 and testosterone deficiency.

After adjusting for all confounding variables, the odds ratios (ORs) for testosterone deficiency in the moderate and high CVH groups were 0.59 (95% CI 0.38–0.92) and 0.38 (95% CI 0.19–0.76), respectively, compared to the low CVH group (**Table 2**). In addition, as shown in **Figure 1**, the RCS analysis results show a negative correlation between LE8 and testosterone deficiency.

**Table 2:** Relationship between LE8 score and testosterone deficiency in the logistic regression models.

	Model 1		Model 2		Model 3	
	OR (95%CI)	*P* value	OR (95% CI)	*P* value	OR (95% CI)	*P* value
LE8 score						
Low (0-49)	Ref		Ref		Ref	
Moderate (50-79)	0.36 (0.24,0.52)	<0.0001	0.37 (0.25,0.55)	< 0.0001	0.59 (0.38,0.92)	0.02
High (80-100)	0.14 (0.08,0.25)	<0.0001	0.15 (0.08,0.27)	< 0.0001	0.38 (0.19,0.76)	0.001
Per 10 points increase	0.63 (0.57,0.71)	<0.0001	0.64 (0.57,0.71)	<0.0001	0.71 (0.61,0.84)	<0.001

Model 1: Adjusted for no covariates.

Model 2: Adjusted for age, race, education and RIP (ratio of family income to poverty).

Model 3: Adjust for the variables in Model 2 plus BMI (body mass index), moderate recreational activity, smoking, alcohol drinking, hypertension, diabetes.

OR, odd ratio.

### Subgroup analysis

We further conducted subgroup analysis of the study population based on age, whose results manifested that the correlation between LE8 score and TD is significantly enhanced among young and middle-aged participants, especially adolescents (**Table 3**).

**Table 3:** Results of subgroup analysis ^a^.

Subgroup	LE8 Score group			*P* for interaction
	Low (0-49)	Moderate (50-79)	High (80-100)	
Age (years)				0.023
20-39	Ref	0.42 (0.14,1.02)	0.10 (0.02, 0.47)**	
40-59	Ref	0.43 (0.20,0.92)*	0.47 (0.18,0.98)	
≥60	Ref	0.90 (0.48, 1.68)	0.94 (0.38,2.11)	

a Each stratification was adjusted for age, race, education, BMI (body mass index), moderate recreational activity, smoking, alcohol drinking, hypertension, diabetes and RIP (ratio of family income to poverty). OR, odds ratio; CI, confidence interval. * means *P* < 0.05, ** means *P* < 0.01.

## Discussion

This research leveraged data from the NHANES in the United States, with the objective of exploring the linkage between LE8 and testosterone deficiency among adult male individuals. The findings reveal a linear relationship between LE8 and testosterone deficiency, where an increase in LE8 score levels is associated with a gradual decrease in the incidence of testosterone deficiency. This relationship holds even after adjusting for other potential confounding factors, where the LE8 score remains significantly negatively correlated with testosterone deficiency. Furthermore, subgroup analysis demonstrated that a significant decrease in the proportion of individuals with testosterone deficiency is primarily observed in the population aged 60 and below.

As the American Heart Association has recently updated its assessment tool for quantifying cardiovascular health (CVH), Life’s Essential 8 (LE8) has emerged as a scoring system that not only offers enhanced sensitivity to individual differences but also highlights the social determinants of maintaining or improving both health and mental health aspects of CVH (10). Currently, the application and value of Life’s Essential 8 are widely recognized as critical indicators for evaluating cardiovascular health (15, 16). Moreover, research has closely linked LE8 to conditions such as chronic kidney disease, non-alcoholic fatty liver disease, and periodontitis (17–19). Yet, research on LE8 and testosterone deficiency remains unexplored. Previous studies have shown that serum testosterone has a direct impact on myocardial and vascular structure and function, as well as on risk factors for cardiovascular disease (20). At present, testosterone exhibits several potential mechanisms through which it can affect cardiovascular risk, including reducing lipoprotein (a), improving cardiac function and aerobic function, promoting coronary vasodilation, reducing fat content, and potentially reducing or delaying the risk of or delay in the development of diabetes (21, 22). In addition, many studies have confirmed that serum testosterone deficiency is an independent risk factor for cardiovascular disease and all-cause mortality (23, 24). A recent Mendelian genetic analysis suggests that genes associated with elevated serum endogenous testosterone levels may also be linked to the risk of myocardial infarction (25). A meta-analysis of drug epidemiology studies in 2018 showed that testosterone treatment can reduce the risk of myocardial infarction (26). Two prospective cohort studies have demonstrated a negative correlation between baseline serum endogenous testosterone concentration and the risk of atrial fibrillation in middle-aged and elderly men (12, 27). Moreover, the relationship between hypogonadism and metabolic disorders is multifactorial and bidirectional, and is associated with disruptions of the hypothalamic pituitary gonadal axis (28). The decrease in serum testosterone levels leads to an increase in lipoprotein lipase expression and an increase in visceral adipose tissue (VAT) accumulation. This accumulation of VAT leads to the release of pro-inflammatory cytokines (TNF-α, IL-1β), and leptin, which directly inhibits the HPG axis and the interstitial cells of the testes, responsible for the production of serum testosterone (29). Additionally, the accumulation of VAT can also lead to an increase in aromatase levels, causing insulin resistance and a reduction in myogenesis via androgen receptor-mediated pathways (30). Considering that previous studies have identified a close correlation between serum testosterone and cardiovascular disease, this emphasizes the necessity of investigating the relationship between the recently updated cardiovascular health measurement method, Life’s Essential 8 (LE8), and the prevalence of testosterone deficiency in adult males in the United States.

There are several reasons that can explain the association between LE8 and serum testosterone. Firstly, LE8 encompasses four health behaviors: Healthy Eating Index (HEI-2015), physical activity, nicotine exposure, and sleep health. Research by Kurniawan et al. revealed that testosterone-related dietary patterns (characterized by frequent consumption of bread and pastries, dairy products, desserts, dining out, and the low-frequency consumption of homemade foods, noodles, and dark green vegetables) are predictive of low testosterone levels and decreased gonadal function (31). Additionally, Hernández-Pérez et al. discovered that sleep deprivation (≤ 6 hours) is associated with higher T concentrations in males, while in middle-aged males (aged 41–64), increased sleep time is linked to lower T concentrations. In women, a type ‘J’ association between sleep time and low T concentration was noted only in the middle-aged category (32). Moreover, Steeves et al. reported that compared to men who engage in 25 minutes or less of physical activity per day, men who participate in more than 65 minutes of physical activity per day experience a significant reduction in the risk of maintaining normal testosterone levels—by half (33). These findings align with the health behavior score of LE8.

On the other hand, the LE8 encompasses four health factors: body mass index, blood lipids, blood sugar, and blood pressure. Hypogonadism frequently accompanies metabolic complications like obesity and diabetes; a reduction or absence of testosterone can precipitate metabolic disorders by elevating visceral lipids and worsening insulin resistance (34, 35). Furthermore, the interplay between hypogonadism and metabolic disorders is recognized as bidirectional, affecting both functional and delayed forms of hypogonadism (29); Hyperglycemia can directly trigger a reduction in testosterone synthesis within Leydig cells by activating the TLR4-mediated oxidative stress pathway (36). Additionally, a high-quality meta-analysis by Brand et al. demonstrated a significant correlation between low testosterone levels and hypertriglyceridemia, noting the greatest degree of association in non-overweight males (37). Thus, the elements included in the LE8 score are intrinsically linked to the risk of serum testosterone levels. LE8 functions as an all-encompassing and readily deployable evaluation instrument in clinical environments, encouraging adherence to healthful behaviors and optimal health metrics. Upholding optimal cardiovascular health (CVH) indicators could represent a viable preventive and management approach to lessen the impact of serum testosterone deficiency and other chronic conditions, including cardiovascular disease.

This study boasts several advantages. Firstly, it utilized a large sample of nationally representative American adults, enabling the research findings to be generalized to a broader population. Secondly, through conducting stratified analysis, it unveiled a strong correlation between LE8 and serum testosterone levels across diverse population groups. However, the current research also faces certain limitations. Firstly, given that too many observations have been excluded, it is challenging to ascertain whether the research data originates from representative samples. Secondly, given the cross-sectional nature of this study, establishing the causal relationship between LE8 and testosterone deficiency proves challenging. Thirdly, the measurement of serum testosterone levels is susceptible to various factors. The current best method for evaluating hypogonadism is to calculate free testosterone. Although rigorous controls were applied during the measurement process, these factors may nonetheless influence the outcomes. Finally, considering the complexity of the sampling design, we used the degree of freedom for measurement data as the number of primary sampling units (PSUs) minus the number of strata, instead of the usual number of observations minus one. This adjustment has a significant impact on the calculation of standard errors and on statistical testing.

## Conclusion

This cross-sectional study demonstrated a negative correlation between the LE8 score and testosterone deficiency. The findings suggest that LE8 offers potential beneficial effects as a viable and effective method for improving serum testosterone levels. To validate these findings, additional prospective longitudinal studies are necessary in the future.

## Data availability statement

The original contributions presented in the study are included in the article/**Supplementary Material**. Further inquiries can be directed to the corresponding author/s.

## Ethics statement

The manuscript presents research on animals that do not require ethical approval for their study.

## Author contributions

MC: Data curation, Methodology, Writing – original draft. JC: Supervision, Validation, Writing – review & editing.

## Supplementary material

Supplementary Figure 1 Flow chart of participants selection.
