# Supplementary figures and images for: Association between life’s essential 8 and testosterone deficiency in men: NHANES 2011–2016
Source: Front Endocrinol (Lausanne). 2024 Jun 3;15:1394383. doi: 10.3389/fendo.2024.1394383 (PMC11180778; doi:10.3389/fendo.2024.1394383)

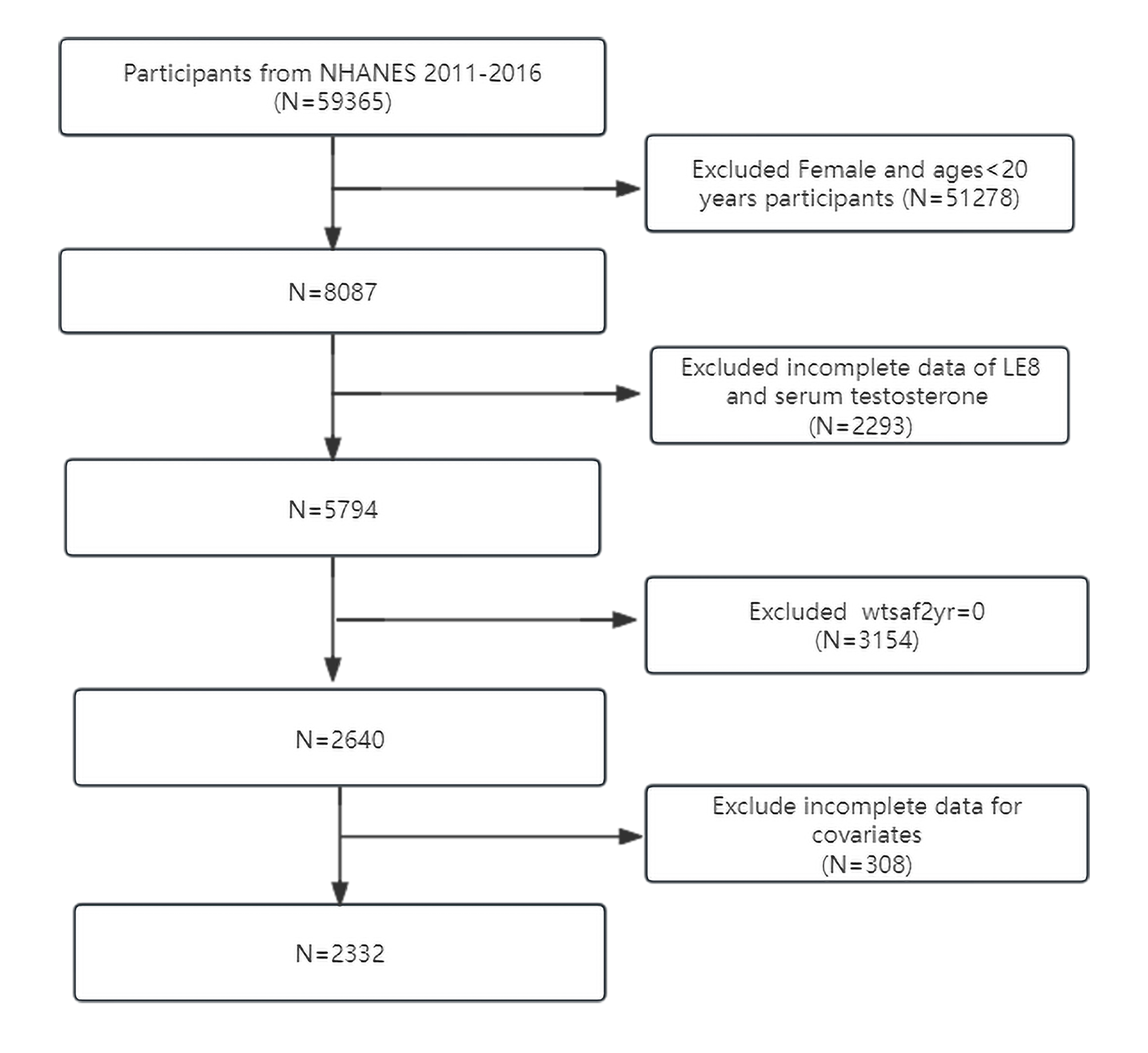

Supplement: Supplementary Figure 1 — Flow chart of participants selection. [file Image_1.tif]
